# Release of Soluble Insulin Receptor From Neurons by Cerebrospinal Fluid From Patients With Neurocognitive Dysfunction and HIV Infection

**DOI:** 10.3389/fneur.2019.00285

**Published:** 2019-03-26

**Authors:** Yamil Gerena, Raissa Menéndez-Delmestre, Andrea Delgado-Nieves, Joyce Vélez, Jarold Méndez-Álvarez, Javier E. Sierra-Pagan, Richard L. Skolasky, Lisa Henderson, Avindra Nath, Valerie Wojna

**Affiliations:** ^1^NeuroHIV Research Program, Department of Pharmacology and Toxicology, School of Medicine, University of Puerto Rico, Medical Sciences Campus, San Juan, PR, United States; ^2^NeuroHIV Research Program, University of Puerto Rico, Medical Sciences Campus, San Juan, PR, United States; ^3^Internal Medicine Residency Program, Damas Hospital, Ponce, PR, United States; ^4^Department of Orthopaedic Surgery, Johns Hopkins University, Baltimore, MD, United States; ^5^Section of Infections of the Nervous System, National Institute of Neurological Disorders and Stroke, National Institutes of Health, Bethesda, MD, United States; ^6^NeuroHIV Research Program, Division of Neurology, Internal Medicine Department, University of Puerto Rico, Medical Sciences Campus, San Juan, PR, United States

**Keywords:** receptor, insulin, tumor necrosis factor, HIV tat protein, insulin resistance, cognitive dysfunction, female

## Abstract

Previously, we found that high levels of soluble insulin receptor (sIR) in the cerebrospinal fluid (CSF) of an HIV-infected women cohort were associated with the presence and severity of HIV-associated neurocognitive disorders (HAND). In this study we investigated if CSF from this population, HIV-1 Tat, and selected cytokines induces sIR secretion from human neuronal cells. Twenty-three (23) HIV-seropositive women stratified by cognitive status and five HIV- seronegative women were evaluated. Soluble IR levels were measured in the extracellular medium of neuronal cells (SH-SY5Y) that were exposed (for 24 h) to the CSF of patients. The levels of sIR, HIV-1 Tat, and cytokine levels (IL-2, IL4, IL-6, IFNγ, TNFα, and IL-10) were quantified in the CSF of participants by ELISA and flow cytometry. Neuronal secretion of sIR was measured after exposure (24 h) to HIV-1 Tat (0.5–250 nM), or specific cytokines. The effects of TNFα and HIV-1 Tat on sIR secretion were also evaluated in the presence of R7050 (TNFα antagonist; 10 nM). Neurons exposed to the CSF of HIV-infected women had higher sIR levels according to the severity of neurocognitive impairment of the participant. Increased CSF sIR levels were associated with the presence and severity of HAND and were positively correlated with CSF HIV-1 Tat levels in HIV-infected women with cognitive impairment. CSF levels of IL-2, IFNγ, and TNFα were significantly increased with HAND. However, only TNFα (5 pg/mL) and HIV-1 Tat (100 nM) induced a significant increase in neuronal sIR secretion after 24 h exposure, an effect that was antagonized when each were combined with R7050. Our data suggests that TNFα and HIV-1 Tat from the CSF of HIV-infected women may regulate the secretion of sIR from neuronal cells and that the effect of HIV-1 Tat on sIR secretion may depend on TNFα receptor activation.

## Introduction

Chronic inflammation in HIV-infected individuals has been associated with insulin resistance, cardiovascular diseases, thrombosis, muscle wasting, neurocognitive impairment, renal and liver diseases, obesity, and premature aging (1–10). Constant production of viral proteins (Tat, Nef, and gp120) and elevated pro-inflammatory cytokine levels (TNFα, IL-1β, IL-6, and IL-8) contribute to persistent neuroinflammation and are associated with cognitive impairment in HIV-infected patients (9, 11–17).

HIV-1 Tat is continuously produced in patients even in the presence of viral suppression, thus promoting the persistence of chronic inflammation in both the peripheral and the central nervous system (18). HIV-1 Tat protein is known to induce TNFα production and high levels of this cytokine are present throughout all stages of HIV infection (19, 20). Although essential for the innate immune response to acute inflammation, persistent elevated levels of TNFα contribute to the maintenance of the chronic neuroinflammation observed in HIV-infected patients. These prolonged high levels of TNFα can lead to glutamate-mediated neuronal toxicity (21) as well as stimulate the release of neurotoxic factors, and cell signaling cascades that lead to neuronal dysfunction and death (13, 20, 22, 23). The combined neuronal damage of viral proteins and cytokines is implicated in the pathogenesis of HIV-associated neurocognitive disorders (HAND) (24–28).

Overproduction of pro-inflammatory cytokines has also been implicated in the pathophysiology of insulin resistance and metabolic disorders (29, 30). TNFα can mediate metabolic abnormalities in different tissues by inducing serine phosphorylation of insulin receptor substrate-1 (IRS-1) protein, decreasing the expression of insulin signaling molecules, and down-regulating the expression of the insulin regulated glucose transporter, GLUT 4 (29, 31, 32). Insulin receptor is expressed on the surface of neurons and its activation can lead to increased neurite growth, gene expression, and energy metabolism. IRS-1 is the major substrate of the insulin receptor (33). Abnormalities in this pathway have been linked with neurodegeneration including Alzheimer's disease (34). However, its role in the pathophysiology of HAND has not been explored.

It is known that people living with HIV (PLH) may develop insulin resistance, HAND, or both, as a result of chronic HIV infection, long-term use of combined antiretroviral therapy (cART), aging, genetic predisposition, or a combination of these factors (35–37). Thus, identification of molecular and cellular pathways linking the high incidence of insulin resistance and HAND in PLH is crucial. Defects in the insulin receptor signaling pathway represents a major target for modulating critical energy functions such as glucose and lipid metabolism, neuronal growth and survival, and synaptic plasticity (38–40). Recently, we showed that high plasma and CSF soluble insulin receptor (sIR) levels are associated with the presence and severity of HAND in a cohort of HIV-infected women on cART (41, 42). Moreover, we observed an increased binding of plasma insulin to sIR in patients with worse cognitive performance (42). Hence, there is a need to identify the factors that contribute to the secretion of sIR into the CSF. In this study, we investigated if the CSF from HIV-infected women with HAND influences the secretion of sIR from human neuronal cells. In addition, we evaluated whether this secretion was regulated by HIV-1 Tat protein, TNFα, or other selected cytokines.

## Materials and Methods

### Participants and Study Design

This was a retrospective cross-sectional study using patient-database information and the sample repository of the Hispanic-Latino Longitudinal Cohort of Women. This study was approved by the University of Puerto Rico Medical Sciences Campus (UPR-MSC) Institutional Review Board, and all the participants signed a written informed consent. This is a unique cohort of Hispanic HIV-infected women characterized longitudinally with viral and immune profiles, neurological exams, and neuropsychological tests. Twenty-four HIV-infected women without a history of diabetes and 5 HIV-seronegative controls were evaluated, as described previously (41–43). Cognitive impairment was determined using the HAND nosology (44). The HIV-infected women were grouped according to their cognitive performance as having normal cognition (NC; *n* = 11), asymptomatic impairment (AI; *n* = 4), and symptomatic impairment [SI, mild neurocognitive disorder (MND) plus HIV-associated dementia (HAD); *n* = 9].

When the HIV-infected women were grouped by cognitive performance, no significant differences were observed between groups with regard to age, body mass index (BMI), current CD4 cell count, CSF and plasma viral load, co-infection with the hepatitis C virus, or toxicology (*p* > 0.05). Most HIV-infected women were using antiretroviral therapy (ART) (82.6%, or 19 of the 23 women) with protease inhibitors (84%, or 16 of the 19 women taking ART). There was no significant difference between groups of increasing HAND severity in either use of ART, protease inhibitor, nor CNS Penetration Effect [using scale prepared by Dr. Scott Letendre (45) (*p* > 0.05) Table 1].

**Table 1:** Characteristics of HIV-infected women.

	**Normal cognition**	**Asymptomatic**	**Symptomatic**	***p*-value**
	***N* = 11**	***N* = 4**	***N* = 9**	
Age (years)	44 (30; 46)	38 (35; 42)	43 (42; 46)	0.123
CD4 (cells/mm^3^)	420.0 (254.8; 573.3)	490.0 (399.5; 588.8)	315.0 (264.5; 474.5)	0.375
CSF HIV RNA (log)	1.70 (1.70; 2.59)	1.98 (1.70, 2.27)	1.70 (1.70, 1.90)	0.562
Plasma HIV RNA (log)	3.12 (1.70; 3.45)	2.73 (1.93; 3.37)	1.76 (1.70, 2.46)	0.126
Hepatitis C virus (% positive)	4/10 (40%)	2/4 (50%)	1/8 (12.5%)	0.335
Body mass index	28.18 (25.9; 32.4)	22.68 (20.95;–)	27.49 (22.43, 30.37)	0.204
Toxicology (% positive)	0/9 (0%)	2/4 (50%); marijuana	2/9 (22.2%); cocaine	0.100
Antiretroviral therapy (% positive)	8/10 (80%)	3/4 (75%)	8/9 (89%)	0.804
Protease inhibitors (% positive)	6/8 (75%)	2/3 (66.7%)	8/8 (100%)	0.278
CNS penetration effect	7 (6; 7)	6 (3; 7)	7 (6; 7)	0.484

*Median (25th, 75th percentile)*.

### Participant's CSF and Neuronal Cells

#### CSF Sample Preparation and sIR Depletion

All spinal taps were performed by the same neurologist (VW) using an atraumatic Sprotte needle. CSF was centrifuged at 130 × g for 10 min at 4°C, and cell pellets and supernatants were separated. Aliquots of supernatant were stored at −80°C. CSF samples tested negative for VDRL. Soluble IR present in the CSF of HIV-infected women and controls were removed prior to incubation with neuronal cells. Aliquots (200 μl) of CSF samples were incubated with anti-hIRβ-specific monoclonal antibody-protein A-Sepharose beads for 16 h at 4°C with gentle agitation. Depleted CSF samples were analyzed using ELISA [27, 29] to confirm the absence of all sIR in the CSF of all the participants.

#### Treatment of Neuronal Cells With CSF

Human neuronal cells (SH-SY5Y; ATCC, Manassas, VA) were grown (5 × 10^5^ cells/well) in 24-well plates supplemented with 10% FBS and 1% penicillin-streptomycin in EMEM growth medium at 37°C and 5% CO_2_. After allowing the cells to adhere overnight, medium was removed and replaced with sIR-depleted CSF (200 μl) from each HIV-infected patient and controls for 24 h. To determine cell viability after incubation with CSF overnight, we performed a MTT assay. Our data demonstrated that cell viability was above 95% after incubation with the CSF (data not shown). The levels of sIR in the CSF were determined by ELISA [27, 29].

#### CSF HIV-1 Tat and Cytokine Levels

HIV-1 Tat levels were measured in the CSF of HIV-infected women (*n* = 20) by sandwich ELISA as described previously (46) at Dr. Avindra Nath's laboratory (NIH-NINDS). Data represent average value of samples run in triplicate on two separate ELISA plates.

The human TH1/TH2 Cytometric Bead Array (CBA) kit (BD Biosciences Pharmingen, San Diego, CA) was used to determine the levels of IL-2, IL-4, IL-6, IL-10, IFNγ, and TNFα in the CSF of HIV-infected women and HIV-seronegative controls. With the CBA, different bead populations with distinct fluorescence intensities had been coated with capturing antibodies specific for different cytokines. After incubation with 50 μL of CSF, the cytokine-captured beads were mixed with phycoerythrin-conjugated detection antibodies to form sandwich immune-complexes. Cytokine levels were performed blinded to group cognitive status and analyzed by flow cytometry.

#### Treatment of Neuronal Cells With Cytokines or HIV-1 Tat Protein

The physiological levels of cytokines in the CSF of HIV-infected women were quantified by flow cytometry, as described above. Cytokines (TNFα: 5 pg/mL, IL-2: 2 pg/mL, IL-6: 10 pg/mL, IL-10: 2 pg/mL, or IFNγ: 2 pg/mL) were added individually to the culture medium of SH-SY5Y neurons for a period of 24 h at 37°C and 5% CO_2_. Cells were also exposed to different HIV-1 Tat concentrations (0.5–250 nM) for 24 h. The secreted sIR levels were analyzed by ELISA. In other experiments, secreted sIR levels were determined after incubation with TNFα (5 pg/ml) or HIV-Tat protein (100 nM) in the presence of R7050 (10^−8^ M).

### Determination of the Soluble Insulin Receptor

Soluble insulin receptor full-length (sIR-αβ, intact) levels methods were previously determined and published (41, 42). Briefly, levels were determined by incubating with an anti-IR ectodomain antibody (1:1,000) (Abcam, Cambridge, MA) for 2 h at 20°C and a FITC-secondary antibody (Abcam, Cambridge, MA). Samples were analyzed in a Cytofluor 4000 (Applied Biosystems, CA) using 485/530 nm excitation/emission filters. The method used to quantify sIR is only capable of detecting intact full-length receptors by ELISA using a capture antibody specific for the amino acids near to the C-terminal of the beta subunit and a primary antibody near to the N-terminal of the alpha ectodomain (41).

### Flow Cytometry

All the flow cytometric analyses were carried out using a FACSCalibur cytometer (BD Biosciences, CA). CellQuest software (BD Biosciences, CA) was used for data acquisition and multivariate analysis. Cells were gated in forward/side scatter dot plots. PE emissions from cytokines were measured in the FL2 (585 nm band-pass filter) channel. Data on scatter parameters and histograms were acquired in log mode. Ten thousand events were evaluated for each sample, and the median peak channel obtained from the histograms was used to determine the levels of CSF cytokines.

### Statistical Analyses

We compared sIR, cytokine, and HIV-1 Tat levels in the CSF of four groups: the control group and the groups of HIV-infected women stratified by cognitive impairment as having normal cognition (NC), asymptomatic impairment (AI), or symptomatic impairment (SI). Because the data were not normally distributed, these comparisons were made using non-parametric analyses. The control group consisted of 5 CSF samples. The use of 5 control samples was adequate to estimate the median and interquartile range. Non-parametric analysis was performed to compare sIR levels secreted from cultured neurons exposed to the CSF of HIV-infected women (stratified by HAND severity) and controls. Parametric one-way ANOVA analysis were used to compare neuronal sIR secretion after exposure to selected cytokines, HIV-Tat protein or combinations with and without R7050. All statistical analyses were performed with IBM SPSS Statistics (version 24) and GraphPad Prism 7. Statistical significance was considered *p* < 0.05.

## Results

### Soluble Insulin Receptor (sIR) Levels in the CSF of HIV-Infected Women

**Figure 1:**
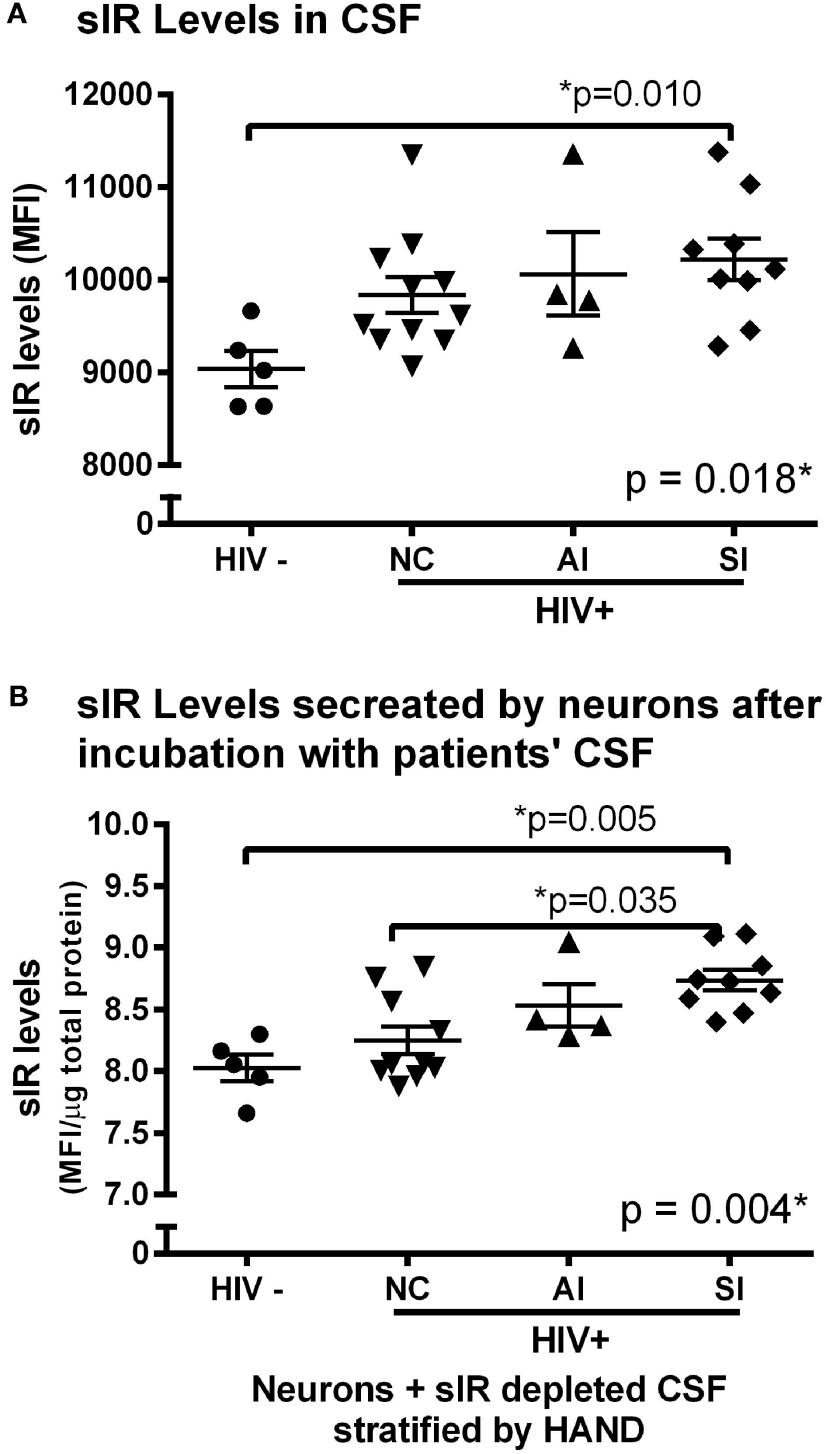
Soluble insulin receptor (sIR) levels in the CSF of patients and neuronal sIR release by neurons after incubation with CSF of HIV-infected women with HAND. **(A)** Higher CSF sIR levels were associated with the presence and increased severity of HAND (HIV-: •, *n* = 5; NC: ▾, *n* = 11; AI: ▴, *n* = 4; SI: ♦, *n* = 9). Scattered dots show the CSF sIR levels of each participant. Horizontal and vertical lines represent mean ± SEM. **(B)** When stratified by HAND severity (NC: ▾, *n* = 11; AI: ▴, *n* = 4; SI: ♦, *n* = 9), higher levels of sIR secreted from neuronal cells were associated with the presence and severity of HAND. Scattered dots show the secreted sIR levels quantified from each CSF participant. Horizontal and vertical lines represent mean ± SEM.

Soluble IR levels were quantified in the CSF of HIV-infected women and HIV-seronegative controls. The levels of sIR were significantly increased in HIV-infected women relative to those of the controls (*p* = 0.002). When HIV-infected women were stratified by HAND, higher levels of CSF sIR were associated with the presence (*p* = 0.018) and increased severity of HAND (*p* = 0.010; Figure 1A). A two-way ANOVA including HAND status, age, and their interaction was used. These covariates did not change the relationship between CSF sIR levels and HAND status.

### sIR Secretion From Neuronal Cells Exposed to the CSF of HIV-Infected Women

Prior to incubating SH-SY5Y neuronal cell culture with the CSF of HIV-seronegative and HIV-infected women, the CSF was successfully depleted of sIR. After 24 h of incubation, sIR secretion was significantly higher in neurons exposed to CSF from HIV-infected women relative to those exposed to CSF from HIV-seronegative controls (*p* = 0.016). When the CSF of HIV-infected women was stratified by HAND, higher levels of full-length sIR secreted from neuronal cells were associated with the presence (*p* = 0.004) and severity of HAND (*p* = 0.035; Figure 1B).

### HIV-1 Tat Levels in the CSF of HIV-Infected Women

**Figure 2:**
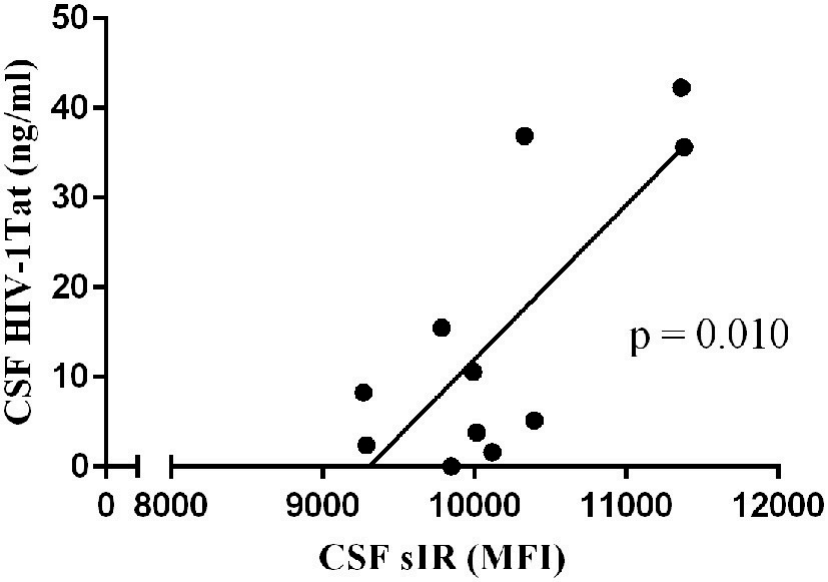
HIV-1 Tat levels in the CSF correlates positively with CSF sIR levels of cognitively impaired HIV-infected women. HIV-1 Tat levels were measured in the CSF of 20 HIV-infected women. When controlling for CSF/plasma ratio of HIV RNA viral load, we observed a significant positive correlation between CSF Tat and CSF sIR among those with symptomatic HAND (•; *n* = 11). Data represent average value of samples run in triplicate on two separate ELISA plates.

HIV-1 Tat levels were measured in the CSF of HIV-infected women (*n* = 20) by sandwich ELISA as described previously (46). CSF HIV-1 Tat levels were undetectable in two of the participants irrespective of their plasma viral load or their HAND diagnostic. When controlling for CSF/plasma ratio of HIV RNA viral load, we observed a significant positive correlation between CSF Tat and CSF sIR among those with symptomatic HAND (*n* = 11; *p* = 0.010; Figure 2).

### Cytokine Levels in the CSF of HIV-Infected Women

**Figure 3:**
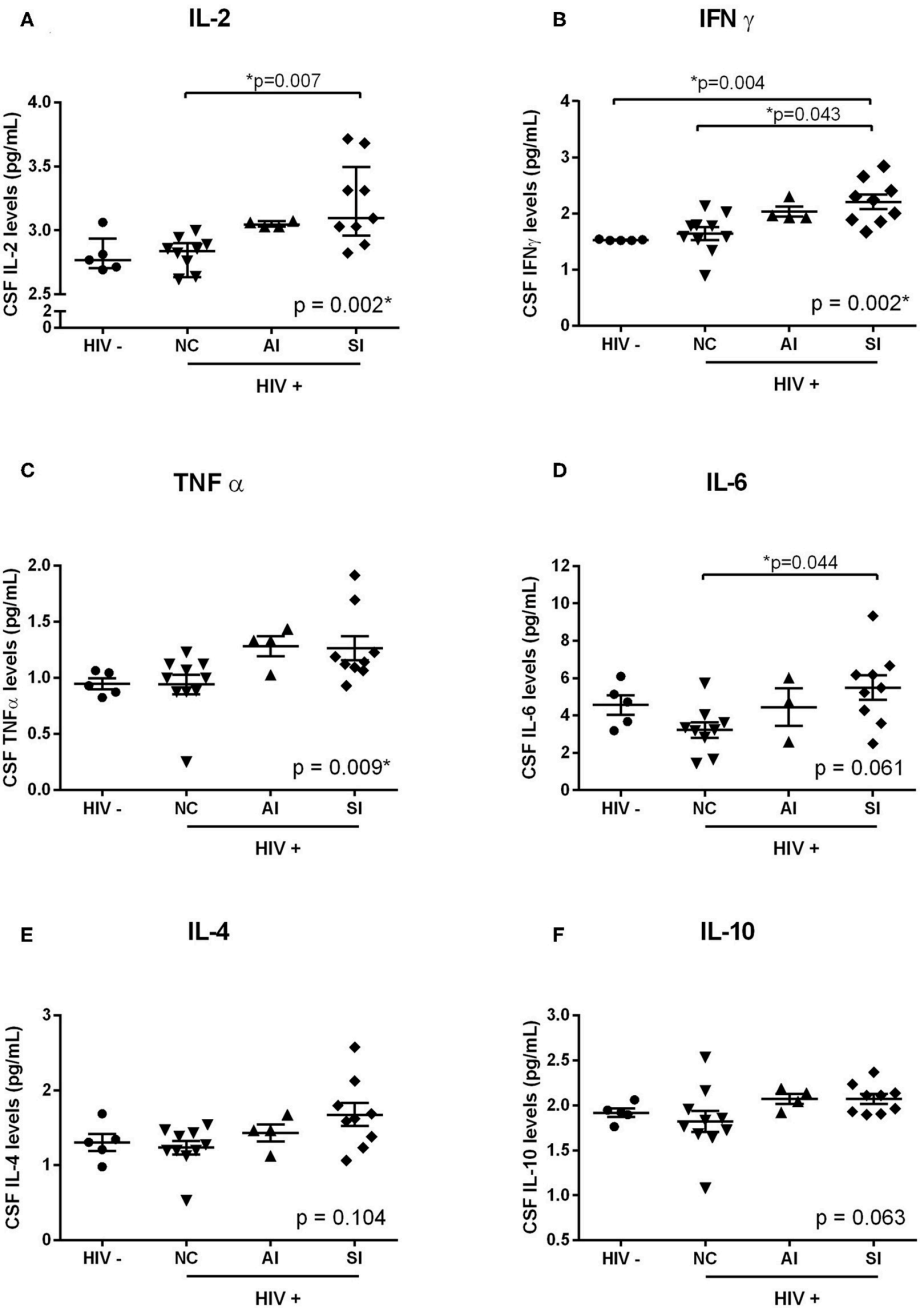
Cytokine levels in the CSF of HIV-infected women. Cytokine levels in the CSF of HIV-infected women (■, *n* = 23; NC: ▾, *n* = 11; AI: ▴, *n* = 4; SI: ♦, *n* = 9) and HIV-seronegative controls (•, *n* = 5) were measured using the human TH1/ TH2 cytometric bead array kit. Significant increased levels of IL-2 **(A)**, IFN-γ **(B)**, and TNF-α **(C)** were associated with the presence and severity of HAND. Although no significant difference was observed with CSF levels of IL-6 **(D)**, a significant difference was observed between HIV-infected women with normal cognition and those with symptomatic neurocognitive impairment in a *post-hoc* analysis. No significant differences were found in the CSF levels of IL-4 **(E)**, or IL-10 **(F)** between HIV-seronegative controls and HIV-infected women (stratified by HAND severity). Scattered dots show the CSF cytokine levels of each participant. Horizontal and vertical lines represent mean ± SEM.

The levels of IL-2, IL4, IL-6, IFNγ, TNFα, and IL-10 were quantified in the CSF of HIV-infected women and controls. Significantly increased levels in the CSF of inflammatory cytokines IL-2 (*p* = 0.002), IFN-γ (*p* = 0.002), and TNF-α (*p* = 0.009) were associated with the presence and severity of HAND (Figures 3A–C). Although no significant difference was observed with CSF levels of IL-6 (*p* = 0.061), a significant difference was observed between HIV-infected women with normal cognition and those with symptomatic neurocognitive impairment in a *post-hoc* analysis (*p* = 0.044, Figure 3D). No significant differences were found in the CSF levels of IL-4, or IL-10 between HIV-seronegative controls and HIV-infected women stratified by HAND (*p* > 0.05, Figures 3E,F). When controlling for CSF/plasma ratio of HIV RNA viral load, we observed no significant correlation between CSF HIV-1 Tat and CSF cytokines. However, a positive correlation was observed between CSF sIR levels and IL-10 (*p* = 0.013; data not shown).

### Neuronal sIR Levels After Cytokine or HIV-1 Tat Treatment

**Figure 4:**
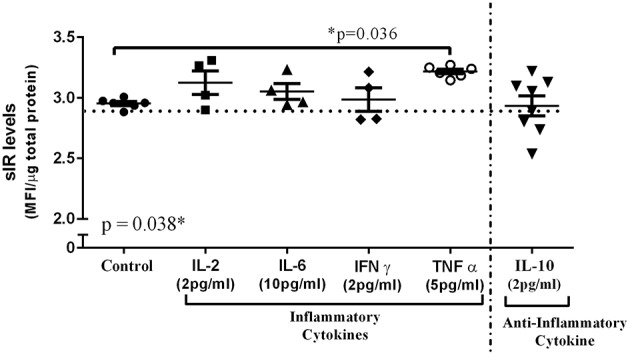
Neuronal sIR levels after incubation with individual cytokines. Cultured SH-SY5Y cells were incubated for 24 h with all elevated cytokines previously quantified in the CSF of HIV-infected patients (IL-2 [■, *n* = 4]; IL-6 [▴, *n* = 4]; IFN-γ [♦, *n* = 4]; TNF-α [○, *n* = 6]; and IL-10 [▾, *n* = 8]). Of these cytokines only TNFα was able to significantly increase the levels of sIR secreted from neurons compared to those that were untreated (•, *n* = 6). Scattered dots show the secreted sIR levels quantified from each well. Horizontal and vertical lines represent mean ± SEM.

Neuronal sIR secretion was measured in the culture medium after 24 h incubation with some of the cytokines previously quantified in the CSF of HIV-infected patients (IL-2, IL-6, IFN-γ, TNFα, and IL-10). Among these cytokines only TNFα was able to significantly increase the levels of sIR secreted from neurons compared to those untreated (*p* = 0.036; Figure 4).

**Figure 5:**
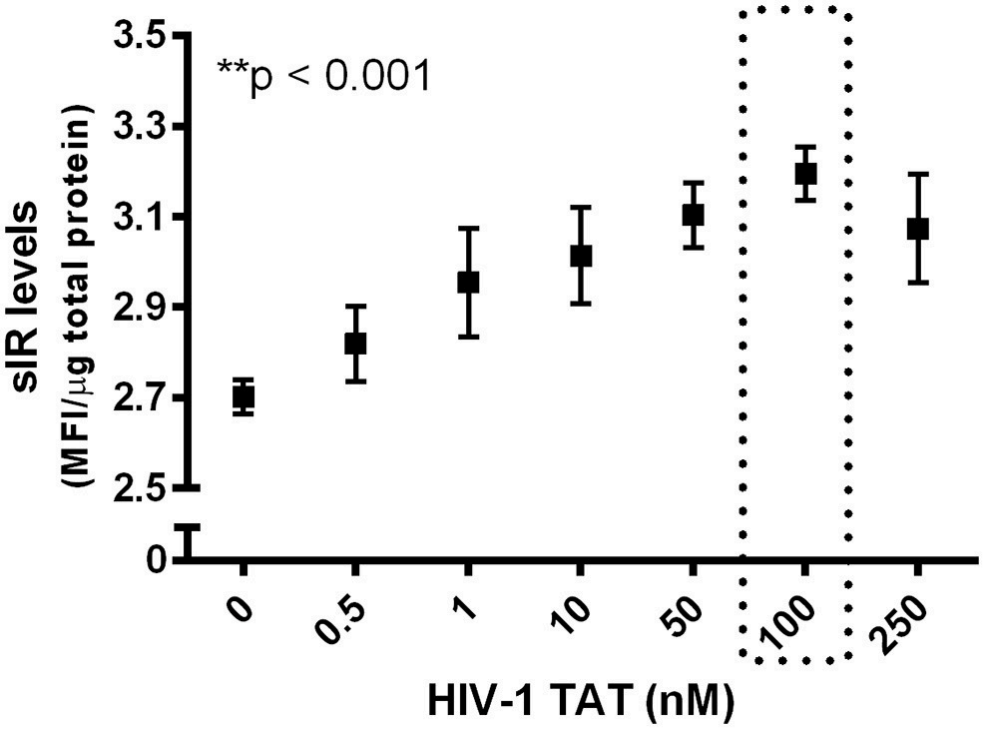
Dose-response curve of HIV-1 Tat vs. sIR levels secreted from neuronal cells. A dose-response curve was created to determine the dose of HIV-1 Tat that would induce maximal sIR secretion (■) after 24 h of incubation. Soluble IR secretion was quantified by ELISA. A concentration of 100 nM induced maximal neuronal sIR secretion (*p* < 0.001). Data on the graph are presented as the mean ± SEM (*n* = 4 for all data points).

We conducted a dose-response curve of HIV-1 Tat (0, 0.5, 1, 10, 50, 100, and 250 nM) to determine the dose that would induce maximal sIR secretion by neurons. We observed a dose-dependent effect of HIV-1 Tat on sIR secretion with maximal response at 100 nM concentration (*p* < 0.001; Figure 5).

**Figure 6:**
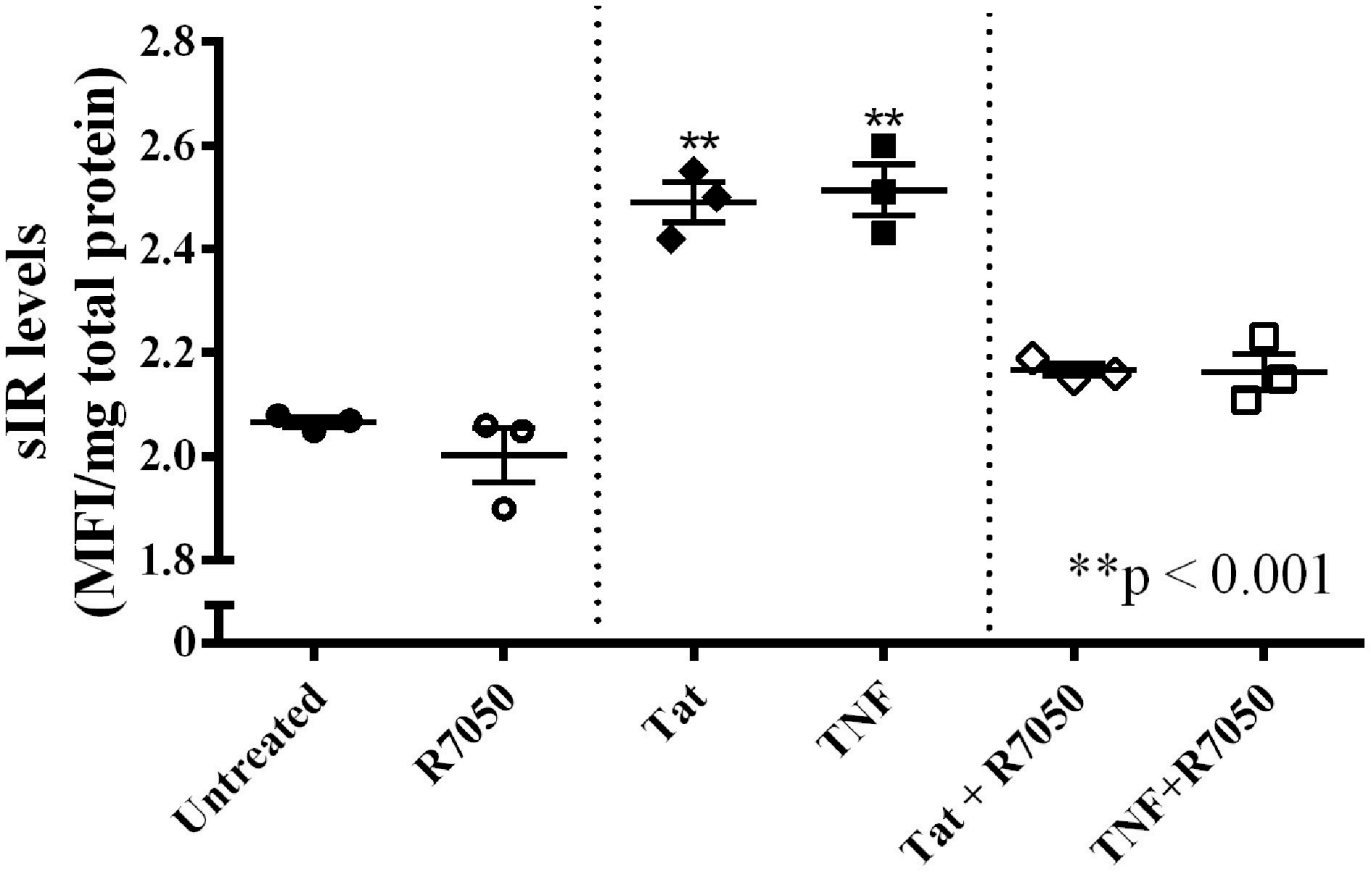
TNFα and HIV-1 Tat induce neuronal sIR secretion through TNFα receptor activation. SH-SY5Y neuronal cells were exposed to HIV-1 Tat (100 nM, ♦); TNFα (5 pg/mL, ■); HIV-1 Tat plus R7050 (10 nM, Δ); or TNFα plus R7050 (10 nM, □) for a period of 24 h. HIV-1 Tat and TNFα increased neuronal sIR secretion significantly (*p* < 0.001) compared to untreated cells (•) or those treated with R7050 (○). However, the introduction of R7050 into the culture medium of cells exposed to HIV-1 Tat and TNFα (Δ and □, respectively) antagonized the sIR secretion induced by both proteins. Scattered dots show the medium sIR levels of each well. Horizontal and vertical lines represent mean ± SEM (*n* = 3 for all groups).

HIV-1 Tat protein is known to induce the production of TNFα in cells derived from brain tissue (44). To determine whether HIV-1 Tat increased neuronal secretion of sIR via activation of TNFα receptors, we incubated the cells for 24 h with HIV-1 Tat (100 nM) or TNFα (5 pg/mL) in the presence of the TNFα antagonist, R7050 (10 nM). Incubation with either HIV-1 Tat or TNFα significantly increased the secretion of sIR by neuronal cells. However, these effects were antagonized by the addition of R7050 to the extracellular medium (*p* < 0.001; Figure 6).

## Discussion

The mechanisms involved in the pathophysiology of insulin resistance and cognitive impairment in HIV-infected participants remain unclear. Several mechanisms may explain the generation of the CSF sIR, including secretion from astrocytes, neurons, or PBMCs that cross the blood-brain barrier (BBB), brain tissue injury, or plasma sIR that crosses the BBB (41). We previously showed that higher levels of plasma and CSF sIR full-length were associated with HAND in a population of HIV-infected women (41). In addition, we observed that plasma sIR served as a scavenger receptor through the binding of higher percentages of insulin in patients with the severe forms of HAND (42). These findings suggest that changes in plasma sIR could be associated with an asymptomatic glucose derangement and may contribute to the development of insulin resistance and HAND (41). The observation of a significant IRS-1 tyrosine phosphorylation decrease suggests the possibility of chronic subclinical cellular insulin resistance, which could contribute to the development of HAND (38).

Although the generation of intact and other sIR isoforms was observed in several cultured cell lines (47), their secretion from neuronal cells has never been explored. In our study we exposed human neuronal SH-SY5Y cells to the CSF of HIV-infected women and found that these cells secreted higher sIR levels compared to the CSF of HIV-seronegative controls. When stratified by cognitive status, this increased secretion was associated with the severity of HAND. The mediators that contributed to this secretion were unknown. Thus, identifying the factors that influence the secretion of sIR to the CSF may help in the development of novel strategies for early intervention and, possibly, the prevention of insulin resistance progression and HAND in this population.

Increasing evidence suggests that inflammation is actively involved in the neuropathogenesis of HAND, and several cytokines have been identified as potential contributors. The importance of these cytokines, especially TNFα, has been emphasized in the propagation and maintenance of a CNS inflammatory response. We were able to quantify six cytokines in the CSF of our cohort of HIV-infected women and observed that the pro-inflammatory IL-2, IFNγ, TNFα, and the anti-inflammatory IL-10 were associated with the presence and severity of HAND. These results are consistent with other studies that show an association between cognitive function and increased levels of IFNγ and TNFα in the CSF of several ethnic populations of people living with HIV (PLH) (13, 48–50). Experimental evidence also shows that higher levels of IL-6 in CSF of HIV-infected patients with HAND confirm its specific role as mediator of neuroinflammation during chronic HIV inflammation (51). In addition, several studies identified possible pathways involved in the generation of IL-6 from brain microvascular endothelial cells, as well as astrocytes (52, 53). Interestingly, when the neuronal cells were exposed to these elevated cytokines, only TNFα was able to induce higher secretion of sIR from neuronal cells. This finding was supported by the fact that R7050, a TNFα receptor blocker, when added to the culture medium for 24 h, antagonized the effects of TNFα in sIR secretion. This is in accordance with previous reports showing that TNFα can induce the secretion of other soluble receptors in the culture medium (54).

Cognitive impairment in HIV-infected patients may be caused directly by the action of the HIV virus or its well-known auxiliary proteins (55). HIV-1 Tat, Rev, Nef, Vif, Vpr, and Vpu can promote neurotoxicity through several mechanisms which result in neuronal dysfunction and cell loss (16, 56). Of these viral proteins, HIV-1 Tat (one of the first HIV proteins to be expressed after infection occurs), has been shown to have a deleterious role in the development and progression of HAND. Indeed, HIV-1 Tat protein can be released from infected cells even in patients who are adequately treated with antiretroviral drugs to affect HIV non-permissive cells such as neurons (18, 57). In our study, we found that HIV-1 Tat increased the secretion of sIR from neuronal cells and R7050 was able to antagonize this effect. The mechanisms employed by HIV-1 Tat on sIR secretion can be explained by its interaction with the toll-like receptor 4 (TLR4). Studies have revealed that HIV-1 Tat interacts physically (with high affinity) with TLR4-MD2 to promote TNFα expression (11). Other studies also show that HIV-1 Tat can induce the secretion of TNFα and IL1β from U87 glioma cells (58). Thus, it is then conceivable that the autocrine effects of TNFα induced by HIV-1 Tat can be conceptualized as a part of the physiological process used by the toxin to regulate neuronal function during HIV infection. Previously we published that sIR acted as a scavenger receptor for insulin promoting intracellular insulin resistance (42). In this study, we observed that the release of sIR is induced by Tat and TNFα suggesting an effect on the insulin signaling pathway and contributing to the neuropathogenesis of HAND. These findings suggest that the replacement of insulin alone such as intranasal insulin may not be sufficient for treatment of these patients.

Our study has several limitations. First, the sample size is small, particularly for the sub-group analyses that we performed. We believe that our sub-group comparisons were sufficiently sized to generate meaningful conclusions; however, our findings should be interpreted with caution. The need to replicate these findings is important. Second, for the purpose of this study we focused on the role of HIV-1 Tat and inflammatory cytokines in the release of sIR from neuronal cells. The presence of glutamate in the CSF and the possible influence of TNF and Tat combination on the secretion of sIR was not measured. Third, we did not adjust for multiple comparisons in our analysis of sIR secretion from neuronal cells. We believe that adjustment was not necessary for two reasons: (1) we conducted planned comparisons that each asked the same basic question, and (2) there was consistency in the results with all comparisons pointing to the same conclusion. Fourth, we only studied a cohort of women with HIV infection since our cohort was designed to study the effects of HIV in women. Further studies to address sex differences are warranted. Fifth, our study design did not consider differentiating the chronic vs. acute effects of HIV-1 Tat. Future studies comparing this effect should be considered.

Soluble receptors, including insulin, may be secreted from cells in several forms such as intact or with an associated cell membrane. Recent studies have observed that the latter form may be secreted as a vesicle as an exosome (59, 60). In this study we proposed to determine the association of the sIR as measured previously in Gerena et al. (41, 42) and inflammation. We understand that further studies including the determination of exosomes should be performed.

Our study provides evidence that the activation of the TNFα receptor plays an important role in the secretion of sIR from neuronal cells. Both TNFα and HIV-1 Tat regulate the secretion of sIR from neuronal cells. Further studies will investigate whether the effects of HIV-1 Tat on sIR secretion are mediated by neuronal TNFα being released to the extracellular medium.

## Author Contributions

YG, RM-D, JV, AN, and VW were responsible for the conception and design of the study. YG, RM-D, AD-N, JM-Á, JS-P, LH, AN, and VW contributed to the data acquisition. RS, YG, RM-D, AN, and VW were involved in the analysis and interpretation of data. YG, RM-D, and VW drafted the first version of the article. All authors revised it critically for important intellectual content. All authors gave final approval of the version to be submitted.

### Conflict of Interest Statement

The authors declare that the research was conducted in the absence of any commercial or financial relationships that could be construed as a potential conflict of interest.
